# LIPOPOLYSACCHARIDE BINDING PROTEIN IS ASSOCIATED WITH LOWER CVD RISK IN OLDER ADULTS

**DOI:** 10.1093/geroni/igz038.3100

**Published:** 2019-11-08

**Authors:** Lisa Roberts, Thomas W Buford

**Affiliations:** University of Alabama at Birmingham, Birmingham, Alabama, United States

## Abstract

Intestinal (i.e. “gut”) permeability may be related to cardiovascular disease (CVD) risk, but biomarkers for gut permeability are limited and associations with CVD risk are unknown – particularly among older adults. This cross-sectional study aimed to determine if serum biomarkers related to gut permeability [intestinal fatty acid-binding protein (iFABP)] and bacterial toxin clearing [cluster of differentiation 14 (CD14), lipopolysaccharide binding protein (LBP)] are associated with CVD risk among older adults. Older adults (n = 38, 72.0 ± 7.1 years old) were stratified by CVD risk category (Adult Treatment Panel: moderate, high-moderate, high). One-way ANOVAs determined differences in each biomarker by risk category, and associations with risk score were evaluated via linear regression and/or Pearson correlations. LBP (p < 0.001), but not iFABP and CD14 (p’s > 0.05), was significantly different between CVD risk categories. Post-hoc tests indicated LBP was higher in the moderate risk compared to both higher risk categories (p < 0.005). LBP was a significant predictor of 10-year CVD risk (β = -0.629; p = 0.001) and evaluation of individual components in the risk score demonstrated a moderate, positive correlation of LBP with total cholesterol (r = 0.326, p = 0.046). Higher circulating concentrations of LBP were associated with lower CVD risk among older adults. Further, total cholesterol was positively associated with circulating levels of LBP. Importantly, cholesterol assists LBP in clearing bacterial toxins from circulation by transporting toxins for removal. These data suggest LBP may be a key component in reducing CVD risk in older adults.

